# The Bioinformatical Identification of Potential Biomarkers in Heart Failure Diagnosis and Treatment

**DOI:** 10.1155/2022/8727566

**Published:** 2022-05-16

**Authors:** Xiaodong Sheng, Xiaoqi Jin, Yanqi Liu, Tao Fan, Zongcheng Zhu, Jing Jin, Guanqun Zheng, Zhixian Chen, Min Lu, Zhiqiang Wang

**Affiliations:** Department of Cardiology, Changshu No. 2 People's Hospital, 18 Taishan Road, Changshu, Jiangsu 215000, China

## Abstract

**Background:**

Heart failure (HF) is defined as the inability of the heart's systolic and diastolic function to properly discharge blood flow from the veins to the heart. The goal of our research is to look into the possible mechanism that causes HF.

**Methods:**

The GSE5406 database was used for screening the differentially expressed genes (DEGs). Gene ontology (GO), Kyoto Encyclopedia of Genes and Genomes (KEGG), and Protein-Protein Interaction (PPI) network were applied to analyze DEGs. Besides, cell counting Kit-8 (CCK-8) was conducted to observe the knockdown effect of hub genes on cell proliferation.

**Results:**

Finally, 377 upregulated and 461 downregulated DEGs came out, enriched in the extracellular matrix organization and gap junction. According to GSEA results, Hoft cd4 positive alpha beta memory t cell bcg vaccine age 18–45 yo id 7 dy top 100 deg ex vivo up, Sobolev t cell pandemrix age 18–64 yo 7 dy dn, and so on were significantly related to gene set GSE5406. 7 hub genes, such as COL1A1, UBB, COL3A1, HSP90AA1, MYC, STAT3 and MAPK1, were selected from PPI networks. CCK-8 indicated silencing of STAT3 promoted the proliferation of H9C2 cells and silencing of UBB inhibited the proliferation of H9C2 cells.

**Conclusion:**

Our analysis reveals that COL1A1, UBB, COL3A1, HSP90AA1, MYC, STAT3, and MAPK1 might promote the progression of HF and become the biomarkers for diagnosis and treatment of HF.

## 1. Background

Heart failure (HF) is defined as the inability of the heart's systolic and diastolic function to properly discharge blood flow from the veins to the heart, resulting in venous stasis and inadequate blood perfusion in the arterial system [1]. In the last few years, HF incidence has been increasing, and the elderly account for a large proportion [2]. According to statistics, 26 million people in the world have been diagnosed with HF, and about 54.35% of them die within 4 years after the diagnosis [3]. Ischemic cardiomyopathy and idiopathic cardiomyopathy are the triggers of HF [4]. Moreover, among HF patients, idiopathic dilated cardiomyopathy (IDCM) has a high mortality rate, and many patients are under 10 years of age. The pathological manifestations of IDCM are left ventricular dilatation, functional contractile failure, and histological manifestations are cardiomyocyte hypertrophy, myofibril loss, and interstitial fibrosis [5]. In addition, IDCM has, approximately, 50,000 hospitalized patients and 10,000 deaths each year, accounting for approximately 25% of all HF cases [6]. Although the survival rate of HF has been improved with the development of medical care, the specific molecular mechanism of HF is still unclear.

The genome-wide gene expression chips are fully combining with present biomedical research. Through the application of planar microfabrication technology and supramolecular self-assembly technology, lots of molecular detection units are integrated on the surface of a tiny solid substrate, which can simultaneously analyze many biomolecules efficiently and quickly at low cost [7]. Genome-wide gene expression chips can not only play a role in early diagnosis, compared with traditional detection methods, they can also detect multiple diseases in multiple patients at the same time on a single chip [8]. Using gene chips, it can also understand the disease at a level [9]. These advantages of gene chips can enable researchers to grasp many diseases' diagnosis information shortly and corresponding therapies [10].

Here, we obtained GSE5406 from Gene Expression Omnibus (GEO) and used GEO2R to systematically determine differentially expressed genes (DEGs). Then, we built a protein-protein interaction (PPI) network and selected the first 7 hub genes through high-level connections. In addition, the Gene Ontology (GO) and Kyoto Encyclopedia of Genes and Genomes (KEGG), as well as the Gene Set Enrichment Analysis (GSEA) pathway analyses, have been performed. Consequently, our results identified 7 hub genes which might have special roles in the course of HF. The hub genes STAT3 and UBB were selected for cell counting kit-8 (CCK-8) cell growth experiment to determine their roles in HF. The study finds a new type of biotargets to assess the diagnosis, treatment, and prognosis of HF.

## 2. Materials and Methods

### 2.1. Microarray Data

GSE5406 is available to the public [11] and includes 210 samples, from which we selected 86 sets of systolic heart failure due to idiopathic dilated cardiomyopathy samples and 16 sets of normally functioning myocardium from unused donor heart samples. Finally, according to the GPL96 platform, we acquired and analyzed GSE5406 matrix files.

### 2.2. DEGs Identification

GEO2R is a website tool for the GEO database based on the R language [12]. We analyzed the DEGs between samples of systolic heart failure due to idiopathic dilated cardiomyopathy and normally functioning myocardium from the unused donor heart. According to the standard, we defined DEGs as the differential expression of FC >1 (the upregulated) and FC <1 (the downregulated). The *P* < 0.0001 was the selection condition to decrease the false-positive rate. In addition, ImageGP was used in hierarchical cluster analysis to display two sets of heat maps.

### 2.3. GO and KEGG Analyses of DEGs

Genes can be annotated through GO and categorized according to biological processes (BP), molecular functions (MF), and cellular components (CC) [13]. KEGG can handle biological pathways and genomes of diseases and drugs [14]. KEGG is basically a tool for a comprehensive and in-depth knowledge of biological systems [15]. We used DAVID (https://david.ncifcrf.gov/) online database, and the CC, MF, BP, and pathways of DEGs were analyzed.

### 2.4. Gene Set Enrichment Analysis

GSEA is a tool issued by the Broad Institute research team of Massachusetts Institute of Technology and Harvard University for the analysis of genome-wide expression profiling microarray data [16]. This study was based on this tool for enrichment analysis of the gene set in the sample.

### 2.5. PPI Network Analysis

PPI, such as physical and functional connections, is evaluated and combined by The Search Tool for the Retrieval of Interacting Genes (STRING) to search for interacting genes [17]. So far, STRING version 10.0 has covered 9,643,763 proteins from 2031 organisms. To estimate the interaction correlation of these DEGs, we used STRING to draw DEGs, and then employed the Cytoscape software for PPI network construction.

### 2.6. Cell Culture

The cell line H9C2 was obtained from the Chinese Academy of Sciences Cell Bank (Shanghai, China), grown in RPMI-1640 (Gibco, USA) supplemented with 10% FBS (fetal bovine serum; Bioind, Israel), penicillin (50 mg/mL) and streptomycin (100 mg/mL, Solarbio, China) in a humid environment with 5% CO_2_ at the temperature of 37°C.

### 2.7. Cell Transfection

We purchased small interfering RNA (siRNA) and negative control (si-NC) from Sangon (Shanghai, China), where siRNA was used to knock down the expression of the hub gene STAT3 and UBB. After that, H9C2 cells were poured into a 6-well plate and transfected with Lipofectamine 2000 (Invitrogen, California) according to the operating steps provided by the manufacturer. After culturing for 48 h, the transfected cells were used for further experiments described in the following sections.

### 2.8. CCK-8 Assay

We performed CCK-8 (Sigma, US) to measure the effect of the hub gene expressions on cell proliferation. 2 × 10^3^ cells/well were put in 96-well plates and cultured for 96 h. In short, 10 *μ*L of CCK-8 solution was added to each well, and the absorbance was measured at 450 nm using a microplate reader.

### 2.9. Statistical Analysis

All experiments were performed in triplicate. The data were presented as the mean ± standard deviation (SD), and SPSS 17.0 (US SPSS Inc.) was used for data analysis. Differences between groups were considered significant for *P* < 0.05.

## 3. Results

### 3.1. Screening of DEGs

**Figure 1:**
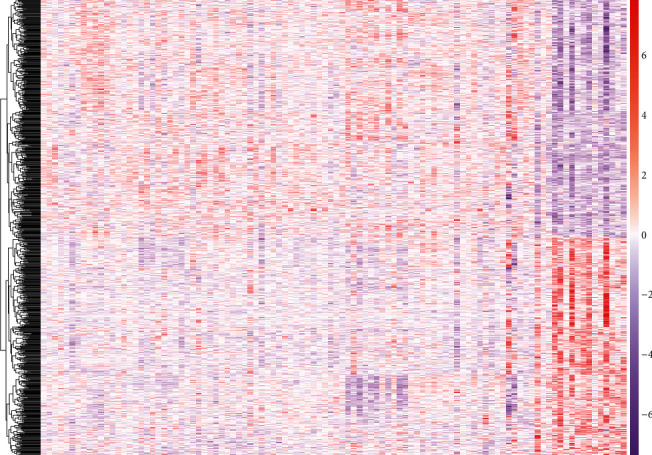
Heat map of all DEGs in HF. Orange represents upregulated DEGs, and purple represents downregulated DEGs.

In this study, 86 groups of systolic heart failure caused by idiopathic dilated cardiomyopathy samples and 16 groups of normal functional myocardium were from unused donor heart samples. *P* < 0.0001 was used as the criterion. After GSE5406 analysis, 838 DEGs were found out, of which 377 were upregulated and 461 were downregulated.  Figure 1 showed the different expressions of these DEGs in 102 samples.

### 3.2. GO and KEGG Enrichment Analysis

**Figure 2:**
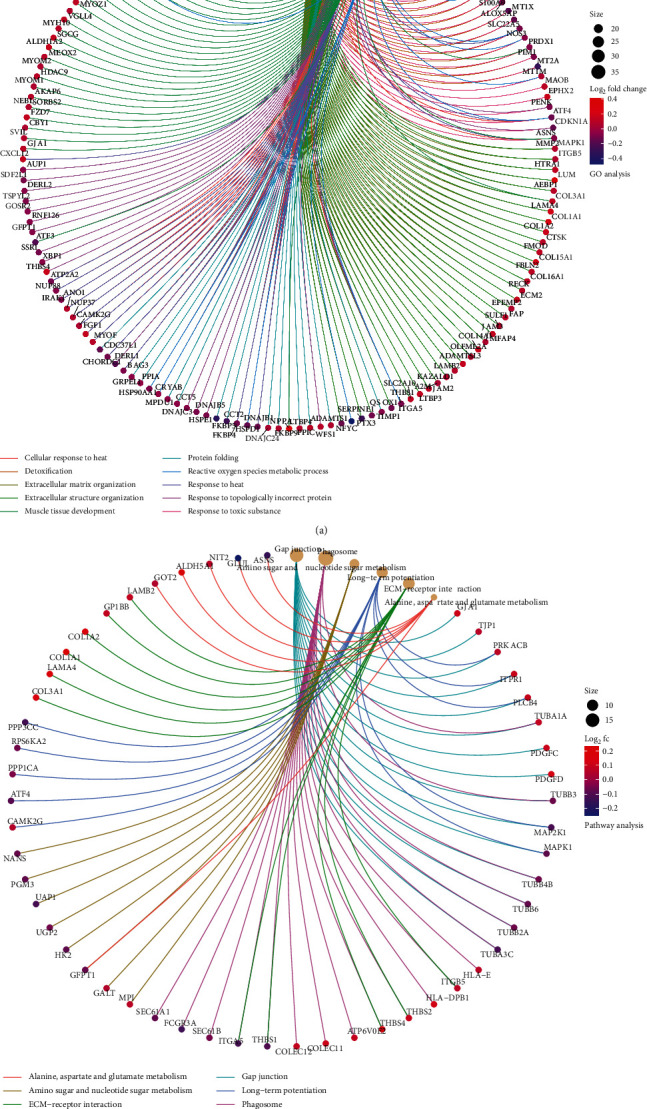
GO and KEGG pathway enrichment analysis. (a) GO enrichment analysis (dots represent genes). (b) KEGG pathway enrichment analysis.

To deeply recognize the selected DEGs, we analyzed GO function and KEGG pathway enrichment. In GO enrichment analysis, DEGs were chiefly related to the cellular response to heat, detoxification, extracellular matrix and structure organization, muscle tissue development, protein folding, reactive oxygen species metabolic process, response to heat, topologically incorrect protein, and toxic substance. In the KEGG enrichment channel, DEGs were concentrated in amino sugar and nucleotide sugar metabolism, alanine, aspartate, and glutamate metabolism, ECM-receptor interaction, gap junction, long-term potentiation, and phagosome signaling pathways. Figures 2(a)-2(b) showed the enrichment maps of GO and KEGG pathways of HF.

### 3.3. GSEA Results

**Figure 3:**
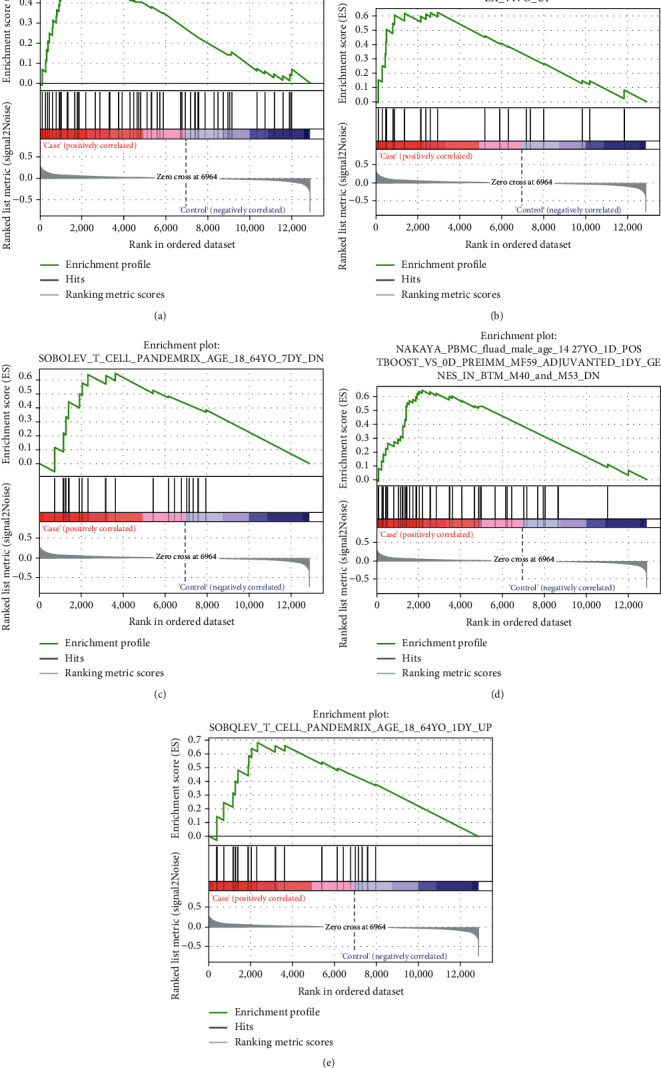
GSEA analysis of pathways related with HF based on dataset GSE5406. The gene sets of (a) Resting vs act cd4 tcell up, (b) Hoft cd4 positive alpha beta memory t cell bcg vaccine age 18–45 yo id 7 dy top 100 deg ex vivo up, (c) Sobolev t cell pandemrix age 18–64 yo 7 dy dn, (d) Nakaya pbmc fluad male age 14–27 yo 1 d postboost vs 0 d preimm mf59 adjuvanted 1 dy genes in btm m40 and m53 dn, and (e) Sobolev t cell pandemrix age 18 64 yo 1 dy up.

After analyzing the GSE5406 gene expression profile through GSEA, we discovered that the gene set in the sample was in resting vs act cd4 tcell up (Figure 3(a)), Hoft cd4 positive alpha beta memory t cell bcg vaccine age 18–45 yo id 7 dy top 100 deg ex vivo up (Figure 3(b)), Sobolev t cell pandemrix age 18–64 yo 7 dy dn (Figure 3(c)), Nakaya pbmc fluad male age 14–27 yo 1d postboost vs 0d preimm mf59 adjuvanted 1 dy genes in btm m40 and m53 dn (Figure 3(d)), and Sobolev t cell pandemrix age 18 64 yo 1 dy up (Figure 3(e)).

### 3.4. Hub Genes Screening

**Figure 4:**
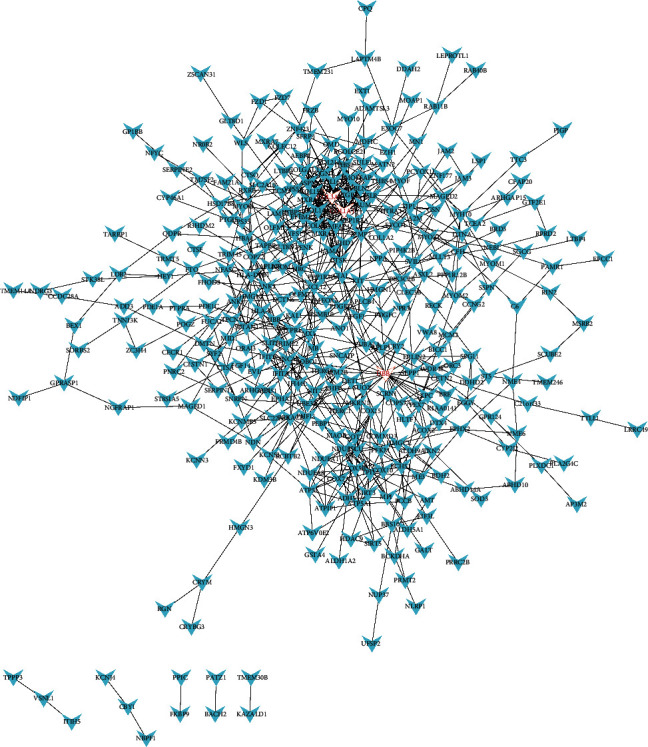
PPI network of upregulated DEGs. Nodes represent genes, and edges represent the connectivity between genes, and the red node represents the hub genes, including 332 nodes and 731 edges.

**Figure 5:**
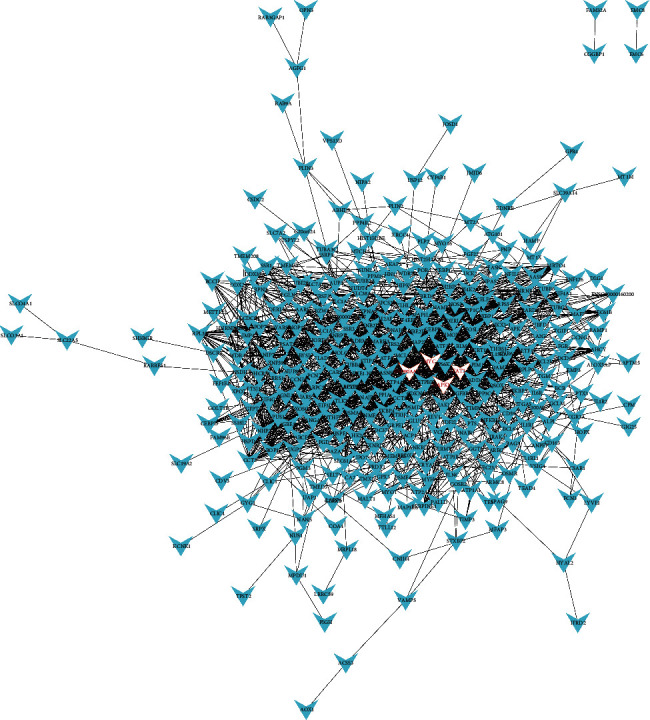
PPI network of downregulated DEGs. Nodes represent genes, and edges represent the connectivity between genes, and the red node represents the hub genes, including 325 nodes and 1495 edges.

We constructed a PPI network of genes according to the connectivity. We screened out 3 hub genes produced by the upregulated DEGs, namely COL1A1 (degree = 33), UBB (degree = 32), and COL3A1 (degree = 31) (Figure 4). Four hub genes were selected from the PPI network map produced by the downregulated DEGs, including HSP90AA1 (degree = 73), MYC (degree = 59), STAT3 (degree = 49), and MAPK1 (degree = 49) (Figure 5). The criteria for selecting hub genes were based on the order of degree from largest to smallest.

### 3.5. Effects of Silencing of STAT3 and UBB on the Proliferation of H9C2 Cells

**Figure 6:**
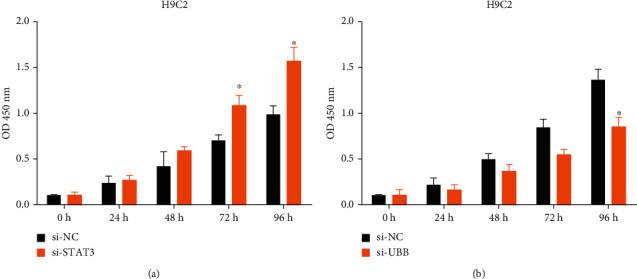
CCK-8 measured the proliferation of the H9C2 cell line. ^*∗*^*P* < 0.05. (a) Cell proliferation experiment graph of downregulation of STAT3 gene. (b) Cell proliferation experiment graph of UBB gene downregulation.

After transfection, we observed the transfection efficiency under a microscope. As shown in Figures 6(a) and 6(b), by using siRNA to downregulate STAT3 and UBB in the H9C2 cell line, we concluded that the cell proliferation rate of the si-STAT3 group was higher than that of the control si-NC. The result of the si-UBB group was that the cell proliferation rate was lower than that of the control group si-NC. It could be seen that UBB was a proto-oncogene, while STAT3 was a tumor suppressor gene.

## 4. Discussion

With the significant aging of the population, HF has become an important social and public health problem [18]. Cardiac remodeling is an important pathophysiological process of heart failure, which is related to the interaction of myocardial cell apoptosis, myocardial hypertrophy and myocardial fibrosis [19]. HF is characterized by a poor prognosis [20]. The prognostic factors of HF are mainly related to age, gender, physical fitness, etiology, ventricular ejection fraction, and cardiac function [21]. HF patient groups have significant differences in etiology, pathophysiology, and natural disease progression [22]. Presently, there are still no effective diagnosis and treatment biomarkers for HF. It is very important to identify promising biomarkers for the diagnosis and prognosis of HF.

Functional and enrichment analyses showed that extracellular matrix organization, ECM-receptor interaction, and Gap junction were the most major signal pathways. Hutchinson et al. found that mechanical stress, inflammation, and oxidative stress all influenced the extracellular matrix (ECM) architecture, which had a highly adaptable structure. The net composition and turnover of ECM are determined by cardiac fibroblasts, muscle, and mast and infiltrating inflammatory cells [23]. Alanine can be used as a fragile indicator and prognostic indicator of all-cause death in elderly patients with HF [24]. Yulong Bao et al. pointed out that the ECM-receptor interaction pathway was the most upregulated gene-rich one [25]. The high expression of ECM protein or gene in HF tissue may provide new ideas for the treatment of HF. Bruce et al. reported that gap junction was a cluster of intercellular channels composed of connexins that mediates the orderly spread of electrical excitement throughout the heart [26]. Previous experiments have suggested that changes in the ventricular gap junction structure and the expression of connexin were the key factors that caused arrhythmia and contractile dysfunction. In the heart, the remodeling of gap junctions, including the reduction of total gap junctions and the downregulation of connexin 43 (Cx 43), leads to the occurrence of arrhythmia [27].

The core DEGs including COL1A1, UBB, COL3A1, HSP90AA1, MYC, STAT3, and MAPK1 were verified as hub genes through PPI networks. The COL1A1 gene is the main component of the extracellular matrix and has been reported in many cancers. Many studies have proved that the expression of COL1A1 plays a certain role in myocardial protection in HF. Hua et al. found that multi-level transcript sequencing identified COL1A1 as a candidate marker for the progression of human heart failure [28]. COL1A1 may be a plasma biomarker of HF and is related to the progression of HF, especially for predicting the progression from HF to 1-year survival rate of transplantation. Dubois et al. pointed out that UBB encoded ubiquitin, which was one of the most conserved proteins known. UBB is a key player in the 26S proteome's breakdown of cellular proteins, related to chromatin structure maintenance, gene expression and stress response. UBB protein is formed by the fusion of a polyubiquitin chain or a single ubiquitin unit with an unrelated protein [29]. Son et al. found that MYC was a proto-oncogene associated with Burkitt's lymphoma (BL), located at chromosome 8q24. MYC (c-MYC) transcription factor is the driving factor of the carcinogenic process. It promotes the elimination of cancer genes by promoting the expression of genes related to cell proliferation. The high expression of MYC promotes the occurrence, development and maintenance of tumors, and is related to aggressive cancer and poor prognosis [30]. Debnath et al. pointed out that signal transducer and activator of transcription 3 (STAT3) could be motivated by multiple cytokines. The loss of STAT3 can easily lead to the development of cardiac fibrosis and other pathogenesis. STAT3, which is specifically lacking in muscle cells, will damage the diastolic function of the heart [31].

Previous studies have told us that there are few studies on the hub gene STAT3 and UBB in HF. Therefore, we selected hub genes STAT3 and UBB for CCK-8 cell proliferation experiments to determine their roles in HF. Through the CCK-8 experiment, we learned about the interference of si-STAT3 on the STAT3 gene reduced its expression in H9C2 cells. The results showed that the H9C2 cell proliferation was more than that of the control group (si-NC), showing that the STAT3 gene was a tumor suppressor gene. After the UBB gene was downregulated, the proliferation ability of H9C2 cell line was lower than that of the control group, indicating that the hub gene UBB played a role of proto-oncogene. In general, STAT3 is the tumor suppressor gene of HF, and UBB is the proto-oncogene of HF. The occurrence of HF is closely related to them and can be used as a relevant target for HF treatment and prognosis.

## 5. Conclusion

In conclusion, our research found 838 DEGs, of which 377 were upregulated DEGs and 461 were downregulated DEGs. We selected 7 hub genes such as COL1A1, UBB, COL3A1, HSP90AA1, MYC, STAT3, and MAPK1, from PPI networks. Among them, STAT3 gene is the tumor suppressor gene of HF, and UBB gene is the proto-oncogene of HF. It may be able to identify significant and new potential targets in carcinogenesis, prognosis, diagnostics in HF by bioinformatics analysis on hub genes and regulatory networks.

## Data Availability

The datasets used and/or analyzed during the current study are available from the corresponding author on reasonable request.

## Abbreviation

HF: Heart failure
DEGs: Differentially expressed genes
GO: Gene ontology
KEGG: Kyoto Encyclopedia of Genes and Genomes
PPI: Protein-protein interaction
IDCM: Idiopathic dilated cardiomyopathy
GEO: Gene Expression Omnibus
GSEA: Gene set enrichment analysis
CCK-8: Cell counting Kit-8
BP: Biological processes
MF: Molecular functions
CC: Cellular components
STRING: Search tool for the retrieval of interacting genes
Cx 43: Connexin 43
ECM: Extracellular matrix
BL: Burkitt's lymphoma
STAT3: Signal transducer and activator of transcription 3.
